# Higher HDL cholesterol levels are associated with increased markers of interstitial myocardial fibrosis in the MultiEthnic Study of Atherosclerosis (MESA)

**DOI:** 10.1038/s41598-023-46811-8

**Published:** 2023-11-17

**Authors:** Omar Chehab, Elie Akl, Ashkan Abdollahi, Ralph Zeitoun, Bharath Ambale-Venkatesh, Colin Wu, Russell Tracy, Roger S. Blumenthal, Wendy S. Post, Joao A. C. Lima, Annabelle Rodriguez

**Affiliations:** 1 Division of Cardiology, Department of Medicine, Johns Hopkins University, Baltimore, MD USA; 2 Department of Radiology, Johns Hopkins University, Baltimore, MD USA; 3 Office of Biostatistics Research, National Heart, Lung, and Blood Institute, National Institutes of Health, Bethesda, MD USA; 4 Department of Pathology and Laboratory Medicine, University of Vermont, Burlington, VT USA; 5 Center for Vascular Biology, University of Connecticut Health, Farmington, CT USA

**Keywords:** Cardiology, Endocrinology

## Abstract

Emerging research indicates that high HDL-C levels might not be cardioprotective, potentially worsening cardiovascular disease (CVD) outcomes. Yet, there is no data on HDL-C's association with other CVD risk factors like myocardial fibrosis, a key aspect of cardiac remodeling predicting negative outcomes. We therefore aimed to study the association between HDL-C levels with interstitial myocardial fibrosis (IMF) and myocardial scar measured by CMR T1-mapping and late-gadolinium enhancement (LGE), respectively. There were 1863 participants (mean age of 69 years) who had both serum HDL-C measurements and underwent CMR. Analysis was done among those with available indices of interstitial fibrosis (extracellular volume fraction [ECV]; N = 1172 and native-T1; N = 1863) and replacement fibrosis by LGE (N = 1172). HDL-C was analyzed as both logarithmically-transformed and categorized into < 40 (low),40–59 (normal), and ≥ 60mg/dL (high). Multivariable linear and logistic regression models were constructed to assess the associations of HDL-C with CMR-obtained measures of IMF, ECV% and native-T1 time, and myocardial scar, respectively. In the fully adjusted model, each 1-SD increment of log HDL-C was associated with a 1% increment in ECV% (*p* = 0.01) and an 18-ms increment in native-T1 (*p* < 0.001). When stratified by HDL-C categories, those with high HDL-C (≥ 60mg/dL) had significantly higher ECV (β = 0.5%, *p* = 0.01) and native-T1 (β = 7 ms, *p* = 0.01) compared with those with normal HDL-C levels. Those with low HDL-C were not associated with IMF. Results remained unchanged after excluding individuals with a history of myocardial infarction. Neither increasing levels of HDL-C nor any HDL-C category was associated with the prevalence of myocardial scar. Increasing levels of HDL-C were associated with increased markers of IMF, with those with high levels of HDL-C being linked to subclinical fibrosis in a community-based setting.

## Introduction

There has been an increasing interest in the link between elevated high-density lipoprotein cholesterol (HDL-C) and worse cardiovascular outcomes^1, 2^. Traditionally, HDL-C was considered to be the “good” or “protective” cholesterol, with numerous observational studies finding that low HDL-C is instead a marker of poor health and worse cardiovascular outcomes^1^. However, numerous randomized controlled trials failed to show any benefit of increasing HDL-C over and above guideline-recommended statin therapy in preventing cardiovascular events^3^. Liu et al. have recently analyzed the association of high HDL-C levels with worsening cardiovascular outcomes using the UK and Emory Cardiovascular biobank^4^. They found a significantly higher risk of all-cause and cardiac-related mortality among participants with HDL-C > 80 mg/dL^4^. These results validated previous reports from population-based cohorts from Copenhagen that also found an increased risk of all-cause mortality among participants with extremely high HDL-C levels^5^. The mechanism of this association of high HDL-C and worse CVD-related outcomes was hypothesized to be related to HDL “dysfunction” rather than “elevation.” HDL-C dysfunction refers to a malfunction in the process of cholesterol efflux capacity^3^. Nevertheless, the focus of elevated HDL-C has been on atherosclerosis, and few studies have investigated the potential mechanisms of HDL-C on other cardiac-related disorders^6^. Cardiac T1 mapping has emerged as a forefront imaging modality to assess for interstitial myocardial fibrosis (IMF), a hallmark of cardiac remodeling that can lead to end-stage heart failure. Recently, Rosmini et al. analyzed the associations of blood lipid constituents with native blood T1 mapping by cardiac magnetic resonance (CMR)^7^. They found that elevated HDL-C in 77 healthy controls was directly associated with an increase in native blood T1 time, a marker closely associated with markers of IMF, commonly indexed also by CMR determined extracellular volume fraction (ECV)^7^. They hypothesized that since women have higher HDL-C levels, this could explain the elevated levels of native myocardial T1 time among women compared to men^7^. Nevertheless, given the very small sample size, it is difficult to draw definitive conclusions from this study.

We, therefore, aim in this work, to utilize the Multi-Ethnic Study of Atherosclerosis (MESA) [2010–2012] to assess the relationship between HDL-C and CMR-markers of diffuse myocardial fibrosis as well as myocardial scar assessed by late gadolinium enhancement (LGE). We hypothesized that compared with those with normal HDL-C levels, participants with low and high HDL-C levels would be associated with greater interstitial fibrosis expressed as longer myocardial native T1 times and ECV, as well as a higher prevalence of LGE-defined myocardial scar.

## Methods

### Study design and participants

MESA is an ongoing longitudinal study of the natural history of CVD in adults and it was established in 2000 with 6814 participants, including White, Black, Chinese, and Hispanic individuals^8^. Men and women aged 45 to 84 years with no prior history of CVD were included from 6 centers (Baltimore City and Baltimore County, Maryland; Chicago, Illinois; Forsyth County, North Carolina; Los Angeles County, California; Northern Manhattan and the Bronx, New York; and St. Paul, Minnesota). The 10-year follow-up of MESA visit 5 was conducted between 2010 and 2012. Among 3012 participants, 1172 had available data and consented to receive CMR contrast agents and undergo T1 mapping for ECV analysis and assessment of myocardial scar using LGE, and 1863 participants had native T1 time available as a measure of IMF. The fifth examination included HDL-C measurements for all participants. Analysis was done among participants who had completed CMR T1 mapping (including ECV and native T1 time) and LGE analysis with available HDL-C measurements. After 10 years of follow-up, 40 participants had a myocardial infarction (MI) as adjudicated by a MESA committee of neurologists, cardiologists, and physician epidemiologists^9^. Informed consent was obtained from all participants at each of the 6 field centers. Institutional review boards approved the study protocol at the Johns Hopkins University Hospital, University of California Los Angeles, University of Minnesota, Wake Forest University Hospital, Northwestern University Hospital, and Columbia University. All methods were performed in accordance with the relevant guidelines and regulations as set by the approving institutions in a standardized manner.

### CMR measures of myocardial fibrosis at MESA visit 5 (2010–2012)

Evidence of increased IMF was defined as an increase in native T1 time and ECV. As for myocardial scar, an exact location of a myocardial scar was characterized by either a focal enhancement in two adjacent short-axis slices or a focal enhancement in one short-axis and a long-axis image. Participants received gadolinium contrast for measuring ECV and assessing the presence of myocardial scar. More participants had available native T1 data since no contrast is required for native T1 analysis and were included in a sensitivity analysis. For those who received gadolinium, a contrast-enhanced magnetic resonance imaging study was conducted on participants with a glomerular filtration rate of 45 mL/min (60 mL/min for participants enrolled at Northwestern University) and no history of allergies to contrast agents, using 1.5 Tesla scanners (Avanto and Espree; Siemens Medical Systems, Erlangen, Germany). The left ventricular (LV) mass, function, and dimensions were determined using cine steady-state free-precession sequencing. Twelve short-axis slices were acquired, along with one four-chamber view and one two-chamber view. Diffuse myocardial fibrosis was assessed as part of the modified Look-Locker inversion recovery (MOLLI) sequence protocol. The MRI protocol used in this study has been described previously^10^. In short, with a single-breath-hold MOLLI sequence, T1 mapping indices were assessed, including pre- (native) and post-contrast T1 times, partition coefficients, and ECV (ECV = 100 × y × [1 − hematocrit]). As a percentage of LV mass, myocardial scars were manually quantified using Medis' QMass software (version 7.2). To define myocardial scars, the area with the most intense signal intensity was manually defined as the full-width at the half-maximum criterion. Prior literature on the prevalence of myocardial scar from MESA was published earlier^11^.

### Statistical analysis

We used STATA 17 (StataCorp LP, College Station, TX) for all analyses. The total cohort was analyzed, followed by stratification by gender, since markers of myocardial fibrosis change differently between men and women with age^10, 12^. A lilliefors test and graphical plots were used to assess the normality of continuous variables. For this analysis, HDL-C was studied as a logarithmically transformed variable and then divided into three categories: 40 mg/dL (low), 40–59 mg/dL (normal), and 60 mg/dL (high), with 40–59 mg/dL as the reference range. Means and standard deviations were used to summarize continuous variables with a normal Gaussian distribution. Categorical variables were presented as counts and relative frequencies (percentages). The linearity of the association between log-HDL-C and ECV and native T1 was assessed using a locally weighted scatterplot smoothing method. Multivariable linear regression was used to evaluate the association between ECV/Native T1 with HDL-C, as a continuous variable and divided into 3 categories (low, normal, high). At MESA visit 5, clinical measures and MRI measurements were included as covariates in the models. In addition, the association between HDL-C and severe IMF, defined as an ECV of more than 30% and native T1 > 954 ms according to Marques et al., was examined using multivariable logistic regression analysis^9^. Additionally, multivariable logistic regression was performed to determine whether HDL-C is associated with myocardial scar prevalence. An analysis of the association between HDL-C and native T1, ECV, and LGE (each separately as the outcome measure) was conducted using four models. The variables selected were based on a theory-driven or hypothesis-driven approach to model building. Model 1 was unadjusted, Model 2 adjusted for age, gender, race, and body mass index, while Model 3 adjusted for the same variables of model 2 as well as LDL-C, triglyceride levels, lipid-lowering medication status, diabetes status, smoking status, systolic and diastolic blood pressure, on antihypertensive therapy, heart rate, eGFR and history of MI. Model 4 included the same variables as model 3 but excluded participants with a history of MI. In this study, statistical significance was defined as a two-sided *P* < 0.05.

## Results

A total of 1863 participants (49% men, mean 69 years) had available HDL-C measurements and had undergone CMR analysis for myocardial fibrosis. ECV quantifications were available in 1172 participants, while native T1 analysis was available in 1863 participants. The study population was 48% White, 14% Chinese, 21% Black, and 15% Hispanic ethnicity. Forty participants with MI events were included in the primary analyses and then excluded in the multivariate analysis model 4. The mean HDL-C level was 55 ± 16 mg/dL, with 267 (14%) participants having low HDL-C (< 40 mg/dl), 988 (53%) with normal HDL-C levels (40–59 mg/dL), and 608 (33%) participants with high HDL-C levels. The mean ECV was within normal limits (27 ± 3), as was the native T1 time (970 ± 46)^19^. Other characteristics of the study cohort are shown in Table 1.

**Table 1:** Baseline characteristics of the MESA study population stratified by HDL-C categories.

Baseline characteristics	Total	HDL-C categories			*P*-value
		< 40 mg/dL	40–59 mg/dL	≥ 60 mg/dL	
Age, years	69 ± 9	67 ± 9	68 ± 9	70 ± 9	< 0.001
Men (n, %)	906 (49)	201 (75)	530 (55)	166 (27)	< 0.001
Women (n, %)	957 (51)	66 (25)	449 (45)	442 (73)	
Body Mass index, kg/m^2^	28 ± 5	30 ± 4	28 ± 5	26 ± 5	< 0.001
Race/Ethnicity (n, %)					
White	902 (48)	112 (42)	471 (48)	319 (52)	< 0.001
Chinese American	270 (14)	32 (12)	157 (16)	81 (13)	
Black	401 (21)	54 (20)	195 (20)	152 (25)	
Hispanic	290 (15)	69 (26)	165 (17)	56 (9)	
Cigarette smoking (n, %)					
Never	848 (46)	106 (40)	452 (46)	290 (48)	0.09
Former	876 (47)	132 (49)	466 (47)	278 (46)	
Current	139 (7)	29 (11)	70 (7)	40 (6)	
Diabetes Mellitus (n, %)	307 (16)	78 (29)	166 (17)	63 (10)	< 0.001
Systolic blood pressure, mm Hg, mean ± STD	122 ± 20	120 ± 18	122 ± 19	122 ± 21	0.2
Diastolic blood pressure, mm Hg, mean ± STD	68 ± 10	68 ± 9	68 ± 10	67 ± 10	0.001
Hypertension medication (n, %)	964 (52)	152 (57)	513 (52)	299 (49)	0.1
LDL cholesterol, mg/dL, mean ± STD	106 ± 32	99 ± 33	107 ± 31	106 ± 30	< 0.001
HDL cholesterol, mg/dL, mean ± STD	55 ± 16	35 ± 3	49 ± 6	74 ± 14	< 0.001
Triglycerides, mg/dL, mean ± STD	110 ± 56	155 ± 70	115 ± 52	83 ± 35	< 0.001
Lipid-lowering medication (n, %)	705 (38)	102 (38)	389 (39)	214 (35)	0.2
eGFR ml/min/1.73 m^2^	80 ± 17	81 ± 18	81 ± 17	79 ± 17	0.3
Myocardial infarction, (n, %)	40 (2)	8 (3)	22 (2)	10 (2)	0.4
Congestive heart failure (n, %)	27 (1.5)	6 (1)	13 (2)	8 (1)	0.5
MRI cardiac measures^†^					
LV end diastolic volume index, mL/m^2^, mean ± STD	64 ± 14	65 ± 14	64 ± 14	64 ± 13	0.4
LV end systolic volume index, mL/m, mean ± STD	24 ± 8	26 ± 9	25 ± 8	24 ± 8	0.01
LV mass/volume ratio, g/ml, mean ± STD	1.04 ± 0.23	1.10 ± 0.27	1.06 ± 0.23	0.99 ± 0.21	< 0.001
LV ejection fraction (%), mean ± STD	63 ± 6	62 ± 6	63 ± 6	63 ± 6	< 0.001
Myocardial native T1 time, msec, mean ± STD	970 ± 46	965 ± 42	967 ± 44	977 ± 49	0.001
ECV, %, mean ± STD	27 ± 3	26 ± 3	27 ± 3	28 ± 3	< 0.001
Blood native T1 time, msec, mean ± STD	1528 ± 89	1515 ± 90	1520 ± 90	1549 ± 84	< 0.001

*ECV* extracellular volume, *eGFR* estimated glomerular filtration rate, *HDL* high‐density lipoprotein, *LDL* low‐density lipoprotein, *LV* left ventricular.

^†^LV volumes and mass are indexed to body surface area.

Table 2 shows the relationship between CMR markers of myocardial fibrosis and log HDL-C. In the regression analyses, log HDL-C was positively associated with both ECV% and native T1 time in the unadjusted and other adjusted analyses (all *p* < 0.05).

**Table 2:** Multivariable association between HDL-C levels with CMR measures of interstitial myocardial fibrosis (extracellular volume fraction and native T1).

Regression models	HDL-C			
	ECV (%) (N = 1172)		Native T1(msec) (N = 1863)	
	β ± SE	*P*-value	β ± SE	*P*-value
Model 1	2 ± 0.3	< 0.001	21 ± 4	< 0.001
Model 2	1 ± 0.3	< 0.001	13 ± 4	0.002
Model 3	1 ± 0.4	0.01	18 ± 5	< 0.001
Model 4	0.9 ± 0.4	0.02	18 ± 5	0.001

Model 1: Unadjusted.

Model 2: Adjusted for age, race/ethnicity, gender, body mass index.

Model 3: Adjusted for variables included in model 2, and lipid-lowering therapy, low-density cholesterol, triglyceride, use of antihypertensive medication, systolic and diastolic blood pressure, diabetes mellitus, smoking status, income, heart rate, estimated glomerular filtration rate, history of MI.

Model 4: Adjusted for variables included in model 3 but excluding those with history of MI.

Table 3 further displays adjusted results for the association of ECV and native T1 with HDL-C after dividing HDL-C into categories (low, normal, and high) with normal levels of HDL-C as reference. In the fully adjusted model, only participants in the high HDL-C category were associated with increased markers of IMF (both ECV [β = 0.5 ± 0.2, *p* = 0.01] and native T1 time [β = 7 ± 3, *p* = 0.01]). Even after excluding participants with MI events, only those in the high HDL-C category were positively associated with markers of diffuse cardiac fibrosis.

**Table 3:** Multivariable association between HDL-C categories with CMR measures of interstitial myocardial fibrosis (extracellular volume fraction and native T1).

HDL-C categories	ECV (%) (N = 1172)								Native T1 (msec) (N = 1863)							
	Model 1		Model 2		Model 3		Model 4		Model 1		Model 2		Model 3		Model 4	
	β ± SE	*P*-value	β ± SE	*P*-value	β ± SE	*P*-value	β ± SE	*P*-value	β ± SE	*P*-value	β ± SE	*P*-value	β ± SE	*P*-value	β ± SE	*P*-value
< 40 mg/dL	− 0.5 ± 0.2	0.04	− 0.2 ± 0.2	0.4	− 0.2 ± 0.2	0.4	− 0.2 ± 0.2	0.4	− 2 ±	0.6	1 ± 3	0.8	− 1 ± 3	0.7	− 1.2 ± 3	0.7
40–59 mg/dL(reference)																
≥ 60 mg/dL	1.01 ± 0.2	< 0.001	0.6 ± 0.2	0.001	0.5 ± 0.2	0.01	0.5 ± 1	0.01	10 ± 2	< 0.001	6 ± 2	0.01	7 ± 3​	0.01​	6 ± 3​	0.02​

Model 1: Unadjusted.

Model 2: Adjusted for age, race/ethnicity, gender, body mass index.

Model 3: Adjusted for variables included in model 2, and lipid-lowering therapy, low-density cholesterol, triglyceride, use of antihypertensive medication, systolic and diastolic blood pressure, diabetes mellitus, smoking status, income, heart rate, estimated glomerular filtration rate, history of MI.

Model 4: Adjusted for variables included in model 3 but excluding those with history of MI.

Tables 4 and 5 show the association between HDL-C and prognostic levels of ECV and native T1 at more than or equal to 30% and 955 ms, respectively. As a continuous variable, HDL-C was positively associated with a higher risk of ECV% ≥ 30% in an unadjusted and mildly adjusted model, but that association became insignificant in a fully adjusted model. However, HDL-C remained significantly positively associated with a higher risk of native T1 time ≥ 955 ms in all models (Table 4). The same relationship was observed when HDL-C was stratified into categories, with the association being significant only among those with HDL-C levels (Table 5).

**Table 4:** Multivariable association between HDL-C levels with CMR measures of interstitial myocardial fibrosis using a cut-off of extracellular volume fraction ≥ 30% and native T1 time ≥ 955 ms.

Regression models	HDL-C (mg/dL)			
	ECV ≥ 30%		Native T1 ≥ 955 ms	
	OR (95% CI)	*P*-value	OR (95% CI)	*P*-value
Model 1	3.12 (1.68–5.79)	< 0.001	2.15 (1.51–3.05)	< 0.001
Model 2	2.14 (1.05–4.34)	0.04	1.50 (0.99–2.26)	0.05
Model 3	2.07 (0.87–4.93)	0.1	2.0 (1.20–3.21)	0.007
Model 4	2.0 (0.8–4.7)	0.1	1.9 (1.2–3.2)	0.009

Model 1: Unadjusted.

Model 2: Adjusted for age, race/ethnicity, gender, body mass index.

Model 3: Adjusted for variables included in model 2, and lipid-lowering therapy, low-density cholesterol, triglyceride, use of antihypertensive medication, systolic and diastolic blood pressure, diabetes mellitus, smoking status, income, heart rate, estimated glomerular filtration rate, history of MI.

Model 4: Adjusted for variables included in model 3 but excluding those with history of MI.

**Table 5:** Multivariable association between HDL-C categories with CMR measures of interstitial myocardial fibrosis using a cut-off of extracellular volume fraction ≥ 30% and native T1 time ≥ 955 ms.

HDL-C categories	ECV ≥ 30%								Native T1 ≥ 955 ms							
	Model 1		Model 2		Model 3		Model 4		Model 1		Model 2		Model 3		Model 4	
	OR (95% CI)	P-value	OR (95% CI	P-value	OR (95% CI	P-value	OR (95% CI	P-value	OR (95% CI	P-value	OR (95% CI	P-value	OR (95% CI	P-value	OR (95% CI	P-value
< 40 mg/dL	1.12 (0.66–1.90)	0.7	1.35 (0.79–2.32)	0.3	1.26 (0.70–2.25)	0.4	1.2 (0.7–2.2)	0.5	0.96 (0.72–1.26)	0.7	1.08 (0.81–1.44)	0.6	1.00 (0.72–1.37)	0.9	1.00 (0.73–1.39)	0.9
40–59 mg/dL (reference)																
≥ 60 mg/dL	1.86 (1.28–2.71)	0.001	1.57 (1.05–2.34)	0.03	1.4 (0.88–2.16)	0.15	1.35 (0.8–2.1)	0.2	1.5 (1.21–1.86)	< 0.001	1.27 (1.00–1.61)	0.05	1.32 (1.02–1.70)	0.03	1.32 (1.02–1.70)	0.03

Model 1: Unadjusted.

Model 2: Adjusted for age, race/ethnicity, gender, body mass index.

Model 3: Adjusted for variables included in model 2, and lipid-lowering therapy, low-density cholesterol, triglyceride, use of antihypertensive medication, systolic and diastolic blood pressure, diabetes mellitus, smoking status, income, heart rate, estimated glomerular filtration rate, history of MI.

Model 4: Adjusted for variables included in model 3 but excluding those with history of MI.

Further subgroup analysis was done after stratifying by sex (Supp Tables 1–4). Among men, increasing levels of HDL-C were associated with higher native T1 times (Supp Table 3). This association was most significant among those in the high HDL-C category. Among women, increasing levels of HDL-C were associated with native T1 time ≥ 955 ms (Supp Table 2).

When analyzing the association between HDL-C categories with blood native T1 time, participants with high HDL-C were associated with a significantly higher native blood T1 time in an unadjusted and mildly adjusted analysis (Supp Table 5). However, this association loses significance in a fully adjusted model.

One thousand one hundred seventy-two participants underwent LGE for assessment of myocardial scar. A total of 106 participants (9%) with myocardial scar (15% men, 2% women) were identified. When analyzing the association between HDL-C and myocardial scar, there was no significant association between HDL-C or any of the HDL-C categories with the prevalence of myocardial scar in adjusted models (Tables 6 and 7).

**Table 6:** Multivariable association between HDL-C levels with prevalence of myocardial scar.

Regression models	HDL-C	
	Prevalence of myocardial scar	
	OR (95% CI)	P-value
Model 1	0.32 (0.15–0.67)	0.003
Model 2	0.8 (0.3–1.9)	0.6
Model 3	1.6 (0.5–5.1)	0.4
Model 4	1.5 (0.5–4.9)	0.5

Model 1: Unadjusted.

Model 2: Adjusted for age, race/ethnicity, gender, body mass index.

Model 3: Adjusted for variables included in model 2, and lipid-lowering therapy, low-density cholesterol, triglyceride, use of antihypertensive medication, systolic and diastolic blood pressure, diabetes mellitus, smoking status, income, heart rate, estimated glomerular filtration rate, history of MI.

Model 4: Adjusted for variables included in model 3 but excluding those with history of MI.

**Table 7:** Multivariable association between HDL-C categories with prevalence of myocardial scar.

HDL categories	Myocardial scar							
	Model 1		Model 2		Model 3		Model 4	
	OR (95% CI)	*P*-value	OR (95% CI	*P*-value	OR (95% CI	*P*-value	OR (95% CI	*P*-value
< 40 mg/dL	1.7 (1.03–2.80)	0.04	1.3 (0.78–2.24)	0.3	1.12 (0.60–2.11)	0.7	1.1 (0.58–2.21)	0.7
40–59 mg/dL (reference)								
≥ 60 mg/dL	0.8 (0.5–1.3)	0.4	1.3 (0.77–2.3)	0.3	1.66 (0.90–3.10)	0.1	1.5 (0.81–2.89)	0.2

Model 1: Unadjusted.

Model 2: Adjusted for age, race/ethnicity, gender, body mass index.

Model 3: Adjusted for variables included in model 2, and lipid-lowering therapy, low-density cholesterol, triglyceride, use of antihypertensive medication, systolic and diastolic blood pressure, diabetes mellitus, smoking status, income, heart rate, estimated glomerular filtration rate, history of MI.

Model 4: Adjusted for variables included in model 3 but excluding those with history of MI.

## Discussion

**Figure 1:**
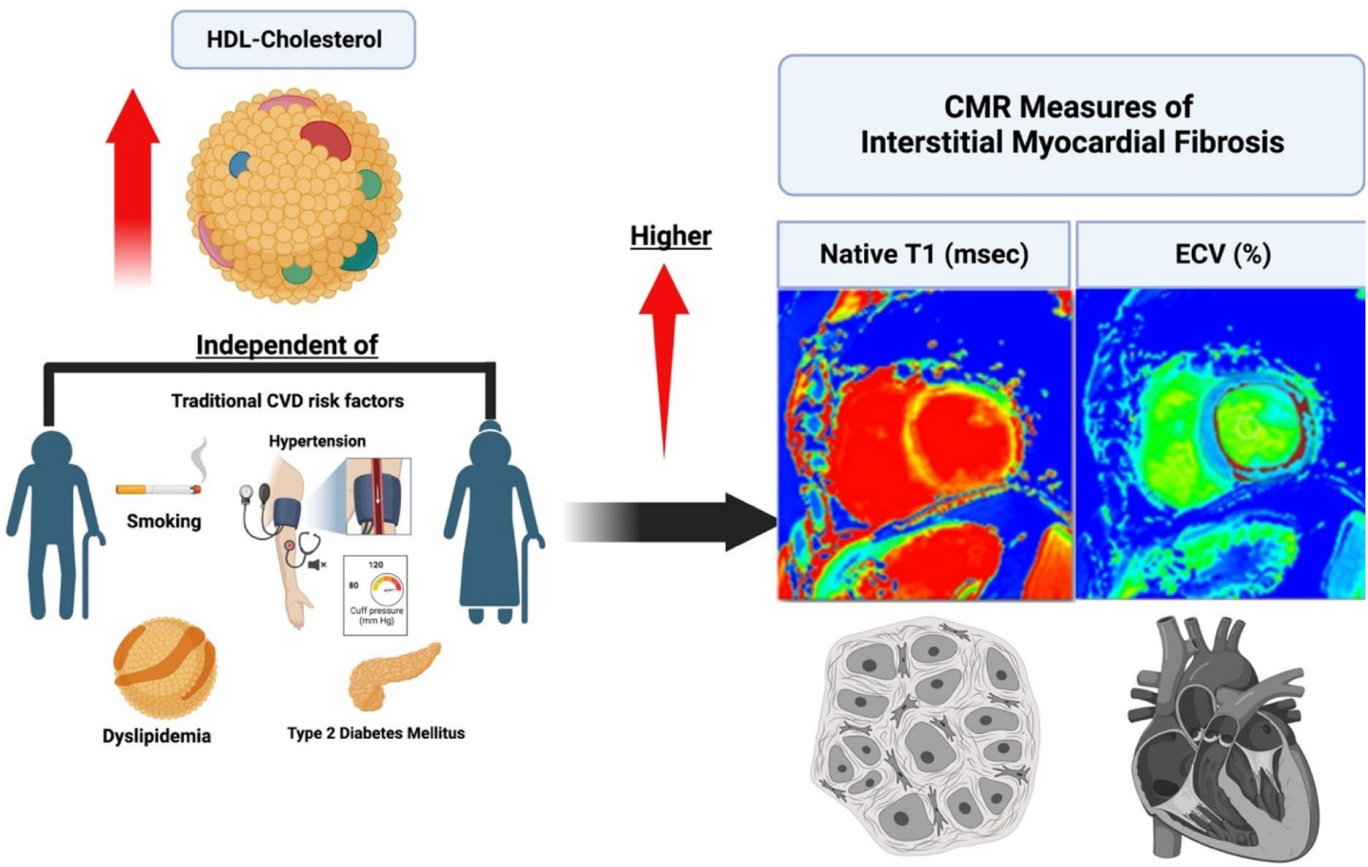
In a community based setting, high levels of HDL-cholesterol were independently associated with increased CMR-markers of interstitial myocardial fibrosis, native T1 and ECV.

In our study, we evaluated the relationship between HDL-C levels with IMF, defined as an increase in native T1 time and ECV percentage, and the prevalence of myocardial scar in a population initially recruited without previous CV conditions. We now show that higher HDL-C levels were positively associated with greater ECV% and native T1 time (Fig. 1). When stratifying HDL-C groups into low, reference, and high categories, our analysis further supported the assessment that high HDL-C is associated with subclinical fibrosis by CMR.

In a previous study, Rosmini et al. studied the effect of blood composition on T1 mapping in a cohort of 77 healthy individuals^6^. They concluded that there was a positive correlation between HDL-C levels and native T1 time in the blood^7^. Given that myocardial and blood T1 are closely correlated, our results support a similar conclusion, specifically that higher HDL-C was associated with increased myocardial native T1 and ECV on the population level. However, in our study, interestingly, native blood T1 time was not associated with HDL-C after adjusting for confounding variables, highlighting a potentially different mechanism between HDL and myocardial T1 analysis. Various conditions affect native T1 values of the myocardium, which will also be reflected in the ECV estimates. Longer native T1 times are associated with tissues where water molecules are less restricted, such as edema or inflammation, or in conditions associated with increased ECV, such as amyloidosis or fibrosis^13^. On the other hand, lower values of T1 are seen in adipose tissues due to slower-moving protons and, thus, shorter T1 recovery time^13^. One explanation for the observed results would be that elevated HDL-C levels affect the relaxation time of nearby photons and therefore affect the native T1 time, similar to the paramagnetic effect of iron in the hemoglobin on T1 relaxation time^14^. Nevertheless, we observed that high HDL-C levels were associated with worse ECV and native T1 levels of 30% and 955 ms, respectively. Recent studies have shown that diffuse myocardial fibrosis evaluated by native T1 and ECV is associated with worse CVD prognosis, increased risk of congestive heart failure hospitalization, and all-cause mortality^9, 15, 16^. A previous analysis from MESA found an ECV and native T1 cutoffs of 30% and 955 ms, respectively, were linked to worse cardiovascular events and mortality^9^. Similar results were observed from the UK Biobank that elevated levels of native T1 were associated with all-cause mortality, worse cardiovascular diseases, and events^12^. However, further work will be needed to elucidate whether the association above reflects a causal contribution of HDL-C in the development of myocardial fibrosis or whether elevated HDL-C levels falsely elevate CMR markers of myocardial fibrosis by affecting T1 relaxation time^7^.

To date, large randomized controlled trials that evaluated drugs such as niacin and treatment that inhibit the cholesterol ester transfer protein such as Torcetrapib have failed to show any signs of efficacy in increasing HDL-C in preventing cardiovascular events^17–19^. Moreover, there is growing evidence in the literature suggesting that elevated HDL-C levels may not offer cardiac protection as previously thought^20^. Several large cohorts found an increased risk of cardiovascular disease with high levels of HDL-C, similar to low levels of HDL-C^20^. A recent study that included two prospective cohorts, the UK and Emory Cardiovascular Biobank, found that compared with those with 40 to 60 mg/dL HDL-C levels, individuals from the Emory Cardiovascular Biobank with very high HDL-C levels (> 80 mg/dL) had a higher risk of all-cause death (1.96 HR, 95% CI 1.42–2.71, *P* < 0.001) and cardiovascular death (1.71 HR, 95% CI 1.09–2.68, *P* = 0.02) after adjusting for confounding factors. Similar results were observed in the UK Biobank and after adjustment of HDL-C genetic risk scores^4^. In another prospective cohort of more than 100,000 participants from the Copenhagen City Heart Study and the Copenhagen General Population Study in Denmark, there was a U-shaped relationship between HDL-C and risk of all-cause mortality, with the high risk being found among low and very high levels of HDL-C^5^. This association was similar in both men and women. Our research findings suggest a potential link between both lower and higher levels of HDL-C and an increased prevalence of myocardial scar. Specifically, individuals with lower HDL-C levels exhibited a slightly elevated risk (OR 1.1, 95% CI 0.58–2.21), while those with higher HDL-C levels demonstrated a moderately increased risk (OR 1.5, 95% CI 0.81–2.89), compared to those with normal HDL-C levels. However, it's important to note that these associations did not reach statistical significance in our analysis.

Our current study found that increased HDL-C was associated with markers of IMF irrespective of sex. However, when stratified by sex, we found that the association was more evident in men; however, an increase in HDL-C was also associated with a significant increase in native T1 in women. Given that women have higher levels of HDL-C, this could be one of the reasons underlying the baseline elevation of IMF markers compared with men^4, 10^.

Another potential theory behind the increase in markers of IMF among those with high HDL-C includes a “loss of function” or “dysfunction” of HDL-C^20^. HDL dysfunction has been found to decrease the ability to promote cholesterol removal from macrophages, prevent LDL oxidation, and control apoptosis, nitric oxide production, monocyte chemotactic protein-1, and vascular cell adhesion molecule expression in endothelial cells. Patients with altered HDL were found to have suppressed nitric oxide production through interaction with LOX-1, TLR2, and TLR4 receptors^20^. This leads to the phosphorylation of inhibitory sites in eNOS instead of activating sites^21^.

Other causes were linked to the inflammatory markers of the lymphocyte activation gene- 3 (LAG-3). Prospective cohorts have found that a decrease in plasma LAG3 protein was one of the causes of the elevation of HDL-C, which was then associated with an increased risk of coronary artery disease^22^. We recently reported that in MESA and the Framingham Heart Study (Offspring cohort) the following plasma proteins were positively associated with LAG3 and HDL-C: IGF1R [insulin-like growth factor 1 receptor], LRIG3 [leucine-rich repeats and immunoglobulin-like domains 3], and DCTPP1 [DCTP pyrophosphastase 1] whereas GFRA1 [glial cell line-derived neurotrophic factor family receptor alpha 1] was inversely associated with HDL-C^23^. Abdellatif et al. examined the role of cardiac IGF1R signaling in an aging model in male mice^24^. These investigators found that young male mice with increased IGF1R signaling exhibited superior cardiac function but this rapidly deteriorated with aging, with decreased autophagic flux and impaired oxidative phosphorylation. For LRIG3, there has been one report of impaired cardiac function and low HDL-C in *Lrig3*^*-/-*^ mice^25^. For DCTPP1 and GFRA1, a PubMed search did not identify any publications linking either protein with HDL-C, myocardial fibrosis, or myocardial scarring. Other possibilities include genetic variants associated with reduced expression of scavenger receptor class B type I protein (SR-BI), which leads to elevated HDL-C and MI risk^20, 26^. Muthuramu et al. showed that expressing hepatic SR-BI in the liver significantly reduced cardiac dysfunction in *Scarb1*^*-/-*^ mice, a mouse model well-known for high HDL-C, accelerated atherosclerosis and coronary artery rupture^26^.

### Limitations

Our study includes several limitations. First, this was a cross-sectional study design; therefore, a causal relationship between HDL-C and CMR measures of myocardial fibrosis cannot be determined. In addition, some factors might affect measures of interstitial fibrosis, such as iron and albumin were not available. It is not clear whether the elevated ECV and native T1 result from an increase in HDL-C or HDL -C alters native T1 time and ECV percentage in a way similar to iron and hematocrit through changes in blood T1. Nevertheless, we showed that pathologically elevated levels of ECV and native T1 were associated with an increase in HDL-C. Even though interstitial fibrosis is the most common determinant of altered T1 indices in this community population, CMR T1 mapping indices are not specific to myocardial fibrosis. A build-up of the extracellular matrix usually occurs because of increased interstitial fibrosis but may also result from edema, hypertrophy, or other cardiac infiltrative disorders. Lastly, those who declined a CMR contrast agent or were not eligible for it could have been systematically healthier than those who were eligible. Thus, temporal or selection bias could not be ruled out. However, even after excluding participants with cardiovascular events such as MI or congestive heart failure, there was a positive relationship between HDL-C and increased CMR markers of interstitial myocardial fibrosis. Nevertheless, CMR T1 mapping and LGE are reliable and noninvasive methods for evaluating myocardial fibrosis with native T1, requiring no contrast injections. This makes it accessible to patients with impaired renal function.

## Conclusion

In a large community-based population, we demonstrated a positive relationship between HDL-C and markers of diffuse cardiac fibrosis. This association seems significant only among those in the high HDL-C category. Whether this relationship reflects a pathologic state of increased inflammation in response to high HDL-C levels warrants further investigation into the mechanism behind HDL-C and its impact on CMR-T1 mapping indices.

### Supplementary Information

Supplementary Tables.

## Data Availability

The data that support the findings of this study are available from MESA committee but restrictions apply to the availability of these data, which were used under license for the current study, and so are not publicly available. Data are however available from the authors upon reasonable request and with permission of the MESA committee.

## Abbreviations

HDL-C: High-density lipoprotein cholesterol
ECV: Extracellular volume fraction
LGE: Late gadolinium enhancement
CHD: Coronary heart disease
CVD: Cardiovascular disease
