# Supplementary material for: Higher HDL cholesterol levels are associated with increased markers of interstitial myocardial fibrosis in the MultiEthnic Study of Atherosclerosis (MESA)
Source: Sci Rep. 2023 Nov 17;13:20115. doi: 10.1038/s41598-023-46811-8 (PMC10656454; doi:10.1038/s41598-023-46811-8)
Supplement: Supplementary file 1 — Supplementary Tables. [file 41598_2023_46811_MOESM1_ESM.docx]

**Supplementary Tables**

**Supplementary Table 1:** Multivariable Association between HDL Cholesterol levels with CMR Measures of Interstitial Myocardial Fibrosis (Extracellular volume Fraction and Native T1) Stratified by Gender

| ​​ | **HDL-Cholesterol**​ | | | | | | | |
| --- | --- | --- | --- | --- | --- | --- | --- | --- |
|  | **Men** | | | | **Women** | | | |
| **Regression Models​** | **ECV (%)​**  **(N=620)​** | | **Native T 1 (ms)​**  **(N=906)​** | | **ECV (%)​**  **(N=552)​** | | **Native T 1 (ms)​**  **(N=957)​** | |
| **​ ​** | **β** ±SE​ | P-value​ | **β**±SE​ | P-value​ | **β** ±SE​ | P-value​ | **β**±SE​ | P-value​ |
| **Model 1​** | 1.6±0.5​ | 0.001​ | 21±6​ | <0.001​ | 1.5±0.4 | <0.001 | 9±6 | 0.1 |
| **Model 2​** | 0.9±0.5​ | 0.06​ | 14±6​ | 0.02​ | 1.3±0.4 | 0.004 | 13±6 | 0.04 |
| **Model 3​** | 0.9±0.5​ | 0.1​ | 21±7​ | 0.002​ | 1.1±0.5 | 0.04 | 19±7 | 0.01 |

Model 1: Unadjusted

Model 2: Adjusted for age, race/ethnicity, gender, body mass index

Model 3: Adjusted for variables included in model 2, and lipid-lowering therapy, low density cholesterol, triglyceride, use of antihypertensive medication, systolic and diastolic blood pressure, diabetes mellitus, smoking status, income, heart rate, estimated glomerular filtration rate, history of myocardial infarction

**Supplementary Table 2:** Multivariable Association between HDL Cholesterol Levels with CMR Measures of Interstitial Myocardial Fibrosis using a Cut-off of Extracellular volume Fraction ≥30% and Native T1 time ≥ 955 msec Stratified by Gender

| ​ | **HDL-Cholesterol**​ | | | | | | | |
| --- | --- | --- | --- | --- | --- | --- | --- | --- |
|  | **Men** | | | | **Women** | | | |
| Regression Models​ | **ECV ≥30 %​** | | **Native T1 ≥955 msec​** | | **ECV ≥30 %​** | | **Native T1 ≥955 msec​** | |
| ​ ​ | OR (95% CI)​ | P-value​ | OR (95% CI)​ | P-value​ | OR (95% CI)​ | P-value​ | OR (95% CI)​ | P-value​ |
| Model 1​ | 4 (1.4-11.6)​ | 0.01​ | 1.6 (0.9-1.8)​ | 0.07​ | 1.7 (0.7-3.8)​ | 0.2​ | 1.7 (1.02-2.9)​ | 0.04​ |
| Model 2​ | 2.7 (0.8-8.9)​ | 0.09​ | 1.1 (0.6-2.1)​ | 0.7​ | 1.8 (0.7-4.3)​ | 0.2​ | 1.9 (1.1-3.4)​ | 0.02​ |
| Model 3​ | 3.6 (0.8-15.7)​ | 0.08​ | 1.5 (0.7-3.1)​ | 0.2​ | 2.2​ | 0.1​ | 2.4 (1.2-4.7)​ | 0.01​ |

Model 1: Unadjusted

Model 2: Adjusted for age, race/ethnicity, gender, body mass index

Model 3: Adjusted for variables included in model 2, and lipid-lowering therapy, low density cholesterol, triglyceride, use of antihypertensive medication, systolic and diastolic blood pressure, diabetes mellitus, smoking status, income, heart rate, estimated glomerular filtration rate, history of myocardial infarction

**Supplementary Table 3:** Multivariable Association between HDL Cholesterol Categories with CMR Measures of Interstitial Myocardial Fibrosis (Extracellular volume Fraction and Native T1 Among Men

| ​ ​ | ECV % | | | | | | Native T1 msec | | | | | | |
| --- | --- | --- | --- | --- | --- | --- | --- | --- | --- | --- | --- | --- | --- |
| ​​ | **Model 1​** | | **Model 2​** | | **Model 3​** | | **Model 1​** | | | **Model 2​** | | **Model 3​** | |
| HDL categories​ | **β** ±SE​ | P-value​ | **β** ±SE​ | P-value​ | **β** ±SE​ | P-value​ | **β** ±SE​ | | P-value​ | **β** ±SE​ | P-value​ | **β** ±SE​ | P-value​ |
| <40​ mg/dL | -0.4±0.3​ | 0.2​ | -0.2± 0.3​ | 0.5​ | -0.3±0.3​ | 0.3​ | 0.8±3​ | | 0.8​ | 3±3​ | 0.4​ | -0.2±4​ | 0.9​ |
| 40-59 mg/dL (reference)​ | | | | | | | | | | | | | |
| ≥ 60​ mg/dL | 0.8±0.3​ | 0.006​ | 0.6± 0.3​ | 0.04​ | 0.5±0.3​ | 0.09​ | 13±4​ | 0.001​ | | 10±4​ | 0.004​ | 11±4​ | 0.005​ |

Model 1: Unadjusted

Model 2: Adjusted for age, race/ethnicity, gender, body mass index

Model 3: Adjusted for variables included in model 2, and lipid-lowering therapy, low density cholesterol, triglyceride, use of antihypertensive medication, systolic and diastolic blood pressure, diabetes mellitus, smoking status, income, heart rate, estimated glomerular filtration rate, history of myocardial infarction

**Supplementary Table 4:** Multivariable Association between HDL Cholesterol Categories with CMR Measures of Interstitial Myocardial Fibrosis (Extracellular volume Fraction and Native T1 Among Women

| ​ ​ | ECV % | | | | | | Native T1 msec | | | | | | |
| --- | --- | --- | --- | --- | --- | --- | --- | --- | --- | --- | --- | --- | --- |
| ​​ | **Model 1​** | | **Model 2​** | | **Model 3​** | | **Model 1​** | | | **Model 2​** | | **Model 3​** | |
| HDL categories​ | **β** ±SE​ | P-value​ | **β** ±SE​ | P-value​ | **β** ±SE​ | P-value​ | **β** ±SE​ | | P-value​ | **β** ±SE​ | P-value​ | **β** ±SE​ | P-value​ |
| <40​ mg/dL | -0.2±0.4 | 0.6 | -0.2±0.4 | 0.7 | -0.1±0.4 | 0.8 | 2±6 | | 0.7 | 0.5±6 | 0.9 | 0.1±6 | 0.9 |
| 40-59 mg/dL (reference)​ | | | | | | | | | | | | | |
| ≥ 60**​** mg/dL | 0.8±0.2 | 0.001 | 0.7±0.2 | 0.008 | 0.5±0.3 | 0.05 | 4±3 | 0.2 | | 5±3 | 0.2 | 5±4 | 0.1 |

Model 1: Unadjusted

Model 2: Adjusted for age, race/ethnicity, gender, body mass index

Model 3: Adjusted for variables included in model 2, and lipid-lowering therapy, low density cholesterol, triglyceride, use of antihypertensive medication, systolic and diastolic blood pressure, diabetes mellitus, smoking status, income, heart rate, estimated glomerular filtration rate, history of myocardial infarction

**Supplementary Table 5:** Multivariable Association between HDL Cholesterol Categories with Native Blood T1 time of Blood

| ​ ​ | Native Blood T1 msec | | | | | |
| --- | --- | --- | --- | --- | --- | --- |
| ​​ | **Model 1​** | | **Model 2​** | | **Model 3​** | |
| HDL categories​ | **β** ±SE​ | P-value​ | **β** ±SE​ | P-value​ | **β** ±SE​ | P-value​ |
| <40​ mg/dL | -5±6 | 0.4 | 8±6 | 0.2 | 10±6 | 0.1 |
| 40-59 mg/dL (reference)​ | | | | | | |
| ≥ 60​ mg/dL | 29±5 | <0.001 | 10±4 | 0.03 | 5±4 | 0.3 |

Model 1: Unadjusted

Model 2: Adjusted for age, race/ethnicity, gender, body mass index

Model 3: Adjusted for variables included in model 2, and lipid-lowering therapy, low density cholesterol, triglyceride, use of antihypertensive medication, systolic and diastolic blood pressure, diabetes mellitus, smoking status, income, heart rate, estimated glomerular filtration rate, history of myocardial infarction
